# Machine learning model for predicting late recurrence of atrial fibrillation after catheter ablation

**DOI:** 10.1038/s41598-023-42542-y

**Published:** 2023-09-14

**Authors:** Jan Budzianowski, Katarzyna Kaczmarek-Majer, Janusz Rzeźniczak, Marek Słomczyński, Filip Wichrowski, Dariusz Hiczkiewicz, Bogdan Musielak, Łukasz Grydz, Jarosław Hiczkiewicz, Paweł Burchardt

**Affiliations:** 1 “Club 30”, Polish Cardiac Society, Warsaw, Poland; 2 Department of Interventional Cardiology and Cardiac Surgery, University of Zielona Góra, Collegium Medicum, 65-046 Zielona Góra, Poland; 3 Nowa Sól Multidisciplinary Hospital, 67-100 Nowa Sól, Poland; 4 Systems Research Institute Polish Academy of Sciences, 01-447 Warsaw, Poland; 5 Department of Cardiology, J. Struś Hospital, 61-285 Poznań, Poland; 6 Faculty of Mathematics and Information Science, Warsaw University of Technology, Warsaw, Poland; 7 Department of Hypertension, Angiology and Internal Medicine, Poznan University of Medical Sciences, 61-848 Poznań, Poland

**Keywords:** Cardiology, Interventional cardiology

## Abstract

Late recurrence of atrial fibrillation (LRAF) in the first year following catheter ablation is a common and significant clinical problem. Our study aimed to create a machine-learning model for predicting arrhythmic recurrence within the first year since catheter ablation. The study comprised 201 consecutive patients (age: 61.8 ± 8.1; women 36%) with paroxysmal, persistent, and long-standing persistent atrial fibrillation (AF) who underwent cryoballoon (61%) and radiofrequency ablation (39%). Five different supervised machine-learning models (decision tree, logistic regression, random forest, XGBoost, support vector machines) were developed for predicting AF recurrence. Further, SHapley Additive exPlanations were derived to explain the predictions using 82 parameters based on clinical, laboratory, and procedural variables collected from each patient. The models were trained and validated using a stratified fivefold cross-validation, and a feature selection was performed with permutation importance. The XGBoost model with 12 variables showed the best performance on the testing cohort, with the highest AUC of 0.75 [95% confidence interval 0.7395, 0.7653]. The machine-learned model, based on the easily available 12 clinical and laboratory variables, predicted LRAF with good performance, which may provide a valuable tool in clinical practice for better patient selection and personalized AF strategy following the procedure.

## Introduction

Atrial fibrillation (AF) is the most common sustained arrhythmia in adults, and its prevalence is increasing^1^. One of the drivers for this increase is the aging population and an intensifying search for undiagnosed AF^2^. The early rhythm-control strategy was associated with a lower risk of adverse cardiovascular conditions in the EAST-AFNET 4 trial^3^.

In this context, catheter ablation is the well-established and most effective treatment option to maintain sinus rhythm^4^. However, the recurrence rate of AF following catheter ablation is common and estimated at 20–45%, which is a significant clinical problem inflating treatment costs^5,6^. One possible explanation for AF recurrence is the complex interaction of various factors such as increasing AF duration, age, left atrium (LA) size, and epicardial fat tissue^7,8^. Our previous study concerning ERAF revealed the poor predictive value of the ERAF model in the patients with abnormal body weight^9^. However, ERAF occurring within the first 3 months following pulmonary veins (PV) isolation by radiofrequency (RF) or cryoballoon approach does not indicate ablation failure, given that the procedure itself generates transient local inflammation. Therefore, the first 90 days following ablation are known as the blanking period^10^.

In contrast, late recurrence (LRAF) occurring 3 months following ablation is considered an actual clinical recurrence, which is a relevant clinical problem^11^. Various machine learning models have been proposed to support predicting LRAF^12–14^. Although deep learning models provide high prediction accuracy^15^, explaining their predictions remains a challenging step, and as stated by the authors of^12^: “we cannot provide an explicit survival function or equation, and we cannot suggest specific cut-off values of predictors because of the ‘black-box’ characteristic of the model”.

In this study, we compared selected top-performing machine learning models for predicting LRAF following PV isolation by cryoballoon or RF ablation. Next, we derived visual explanations using the well-known SHapley Additive exPlanations (SHAP) framework^16^. SHAP enables us to assign an importance value for each feature in a particular prediction. Understanding why the model makes a certain prediction is as important as the accuracy of developed models. Concluding, similarly to Kim et al., we considered ERAF as an explanatory variable, and the major contribution of this work is confirming that ERAF is an important predictor of LRAF^10^.

## Methods

### Study population

This study comprised 201 consecutive patients with documented symptomatic paroxysmal, persistent, and long-standing persistent AF. The patients were scheduled to undergo cryoballoon and RF ablation using the CARTO-mapping at the Cardiology Department in the Multidisciplinary Hospital in Nowa Sól, Poland. A total of 164 patients underwent the PV isolation procedure for the first time, while 34 and 3 patients underwent ablation for the second and third time, respectively^9^. Exclusion criteria included intracardiac thrombi, myocardial infarction, stroke or cardiac surgery in the previous 3 months, malignancies, autoimmune or inflammatory disease, antibiotic therapy, and heart failure exacerbation. All the patients signed a written study participation consent while the study protocol was approved by the Medical Ethics Committee at Poznań University of Medical Sciences (Approval 44/16). The study was carried out in May 2016 until March 2018^9^. All the participants fulfilled the criteria and completed the study.

### Radiofrequency ablation

Pre-procedural transthoracic and transoesophageal echocardiography (TEE) were performed in all the patients prior to ablation. RF ablation was performed using the focal ablation strategy guided by the CARTO 3-D mapping system (Biosense Webster, Diamond Bar, CA). The ablation procedure was performed under local anesthesia with mild conscious sedation. The double transseptal puncture with LassoNav and Navistar ST electrodes was performed following the fluoroscopic guidelines. Immediately after the puncture, intravenous unfractioned heparin (UFH) was administered. An activated clotting time was maintained between 300 and 350 s^11^. The voltage map of left atrium and PVs was performed. PV isolation was performed using 7F Navistar ThermoCool and 8F ThermoCool SmartTouch SF (Biosense Webster, Diamond Bar, CA). The standard energy settings were 30 Watts for 30 s at the anterior LA wall, and 20 Watts at the posterior LA wall. In 5 patients, RF ablation was performed using the “ablation index” algorithm^9^. The verification of the lines was done using the voltage map. Additional cavotricuspid isthmus ablation was performed in the patients with a concomitant typical atrial flutter. Additional ablations such as low-voltage zone ablation, complex fractionated atrial electrogram-guided ablation, or linear ablation were performed at the operator’s discretion if AF was induced after PV isolation.

### Cryoballoon ablation

All the procedures were performed under local anesthesia with mild conscious sedation. In the cryoablation group, the second-generation cryoballoon ablation catheter was used (Arctic Front Advanced, Medtronic, Minneapolis, MN, USA). The patients had a groin entry venous route catheter introduced with the transseptal puncture by means of a Brockenbrough needle (St. Jude Medical). In addition, a 15 Fr steerable sheath (FlexCath Advance, Medtronic) and an integrated inner-lumen circular mapping catheter (CMC, Achieve™; Medtronic, Inc.) were applied^9^. The cryoballoon was introduced into the target PV, and its position was confirmed by contrast retention. The freezing cycles, lasting 180–240 s, were performed for each PV and were confirmed by the Achieve catheter^9^. In the absence of PV potentials, the procedure was ended; otherwise, next cryoapplications were performed. During the application in the right veins, the diphragmatic nerve was constantly stimulated (30/min) to avoid its paralysis. Freezing was immediately terminated at any indication of diaphragmatic weakness or palsy.

### Biochemical analyses

Blood samples were obtained at baseline and 24 h after ablation^9^. Venous blood was drawn from the basilic vein. All routine biochemical analyses (hsTnT, CK, CKMB, CRP, D-dimer, fibrinogen) were performed immediately in the central hospital laboratory. High-sensitivity TnT (hsTnT) was analyzed by electrochemiluminescence immunoassay (ECLIA) The principle of the Sandwich ECLIA method involves the immobilization of Troponin T using a biotinylated monoclonal anti-Troponin T antibody and a monoclonal anti-Troponin T antibody labeled with a ruthenium complex. HsTnT were measured by means of a Cobas c601 device with a cut-off value of 14 pg/L (Roche Diagnostics GmbH, Germany). The serum creatinine level was measured using Creatinine Jaffe Gen.2 kits (CREJ2; Roche, Mannheim, Germany) based on a kinetic colorimetric assay. This kinetic colorimetric assay is based on the Jaffé method. In alkaline solution, creatinine forms a yellow-orange complex with picrate. The rate of dye formation is proportional to the creatinine concentration in the specimen. The levels of sodium and potassium were determined by indirect potentiometry using ion-selective electrodes (ISE) (COBAS C501, Roche, Germany). Serum aspartate aminotransferase (AST) and alanine aminotransferase (ALT) were determined by means of a kinetic method with NADH and TRIS buffer (Roche, Mannheim, Germany). CK was marked using a kinetic serum test with fosfocreatine and ADP. Creatine kinase (CK) catalyzes the reaction between creatine phosphate (CP) and adenosine 5′-diphosphate (ADP) with formation of creatine and adenosine 5´-triphosphate (ATP). The latter phosphorylates glucose to glucose-6-phosphate (G6P) in the presence of hexoquinase (HK). G6P is oxidized to Gluconate-6P in the presence of reduced nicotinamide-adenine dinucleotide phosphate (NADP) in a reaction catalyzed by glucose-6-phosphate dehydrogenase (G6P-DH). The conversion is monitored kinetically at 340 nm by the rate of increase in absorbance resulting from the reduction of NADP to NADPH proportional to the activity of CK present in the sample. CKMB was analyzed with CKMB immunoassay concentrations (Roche, Germany). The test contains two monoclonal antibodies against epitopes of the CK‑MB molecule, one gold-labelled, the other biotinylated. The antibodies form a sandwich complex with CK‑MB in the blood. CRP was tested with an immunoturbimetric latex CRP assay (Roche Diagnostics GmbH). Human CRP agglutinates with latex particles coated with monoclonal antiCRP antibodies. The precipitate is determined turbidimetrically. D-dimer assays were inspected with an immunoturbidimetric method using STA-Liatest D-Di Plus (Stago, France). The assay was calibrated with the calibrator of the manufacturer. Fibrinogen, INR, APTT were measured by STACompact Max mechanical coagulometer (Diagnostica Stago, France). The STA Compact Max analyser’s method of measuring the coagulation time is based on the mechanical registration of the viscosity based detection system (VBDS). In the analysis, the peripheral blood count was marked with CELL-DYN Ruby using flow cytometric techniques combined with the MAPSS technology (Abbott Diagnostics, USA)^9^. In the study, residual fresh (< 4 h) ethylenediaminetetraacetic acid (EDTA)-anticoagulated samples were used. Normal reference ranges were as follows: WBC, 4.0–10.0 (× 10^9^/L), Fibrinogen 200–400 mg/dl; CK 0–190 U/L and CK-MB 7–25 U/L. The CRP and D-dimer cut-off values were 0.5 mg/dl and 0.5 μg/ml respectively. The extent of biomarker elevation was defined as the post-procedure recorded value minus the baseline value (day 0).

### Clinical follow-up

The patients were monitored for the first 24 h following ablation. The 24-h Holter monitoring was installed in an outpatient clinic within the first 3 months after ablation and every 6, 9, and 12 months during the follow-up (Mortara Instrument, Milwaukee, WI). Additionally, a 12-lead electrocardiogram (ECG) was recommended for the patients with the symptoms of arrhythmia. LRAF was defined as any symptomatic or asymptomatic atrial tachyarrhythmias (AF, atrial tachycardia [AT], or atrial flutter [AFL]) lasting > 30 s that occurred from 3 months to 1 year). Antiarrhythmic drugs (AAD) were not routinely used after ablation, except for the highly symptomatic patients with ERAF. Oral anticoagulants were continued for at least 2 months^11^. The decision to continue anticoagulation was based on the individual's stroke risk determined by the CHA2DS2-VASc score^9,11^.

### Statistical analyses

We considered the following two groups of patients depending on the occurrence of LRAF: (1) patients with LRAF; (2) lack of LRAF. The normal distribution of continuous variables was tested using the Shapiro–Wilk test. Next, the Mann–Whitney test was used for not normally distributed variables, and the Student's *t*-test was used for normally distributed variables. Also, the Pearson chi-square test for independence was applied for categorical variables. The analyses were done using the R programming language. The statistical threshold for significance for *p* values was 0.05.

### Model development for AF prediction

We formulated LRAF as a binary classification problem and predicted its occurence. In the experimental evaluation, we adapted the following top-performing benchmark machine learning algorithms: random forest (RF), decision trees (DT), support vector classifier, XGBoost (XGB), and logistic regression (LogR). The classifiers were constructed using the sklearn and XGBoost libraries from the Python programming language. Finally, SHAPley values were calculated to explain LRAF predictions. SHAP is one of the most frequently used model-agnostic methods and is commonly used for tabular data^16^. SHAP explanations were derived for the top-performing classifier, namely XGBoost, using the SHAP library for Python.

*Experimental Set-up* The dataset with all the patients was randomly split into training and test sets (90%) and a validation set (10%). Next, the repeated stratified fivefold cross-validation was applied to train the classification algorithms for the training set and select the optimal subsets of variables to be included in the predictive model. The permutation importance algorithm (with a number of permutations = 50) was applied to reduce dimensionality and select the subset of variables with the following indices i = 8, 12, 16, and 20. The subset of variables was considered optimal if the F1 score for the test set was maximal. Also, the HAS-BLED score (a scoring system developed to assess 1-year risk of major bleeding) was added to the subset of selected variables to improve the interpretation potential of the model outcomes. For a fair comparison, the same subsets of data were considered for each fold, regardless of the algorithm.

### Ethics approval and consent to participate

The study was conducted according to the guidelines of the Declaration of Helsinki, and approved by the Ethics Committee of Poznan University of Medical Sciences (protocol code 44/16). Signed informed consent was obtained from every subject involved in the study.

## Results

The study comprised 201 patients with AF treated with cryoablation (122 patients) and RF ablation (79 patients). Over 80 baseline clinical, procedural, and laboratory characteristics, stratified by the presence of LRAF during the follow-up, which were considered in this study are described in Table S1 in Supplementary Materials. Their statistical characteristics are summarized in Table 1. Additional laboratory data are presented in Table S2 in the Supplementary Materials. During the first year, LRAF occurred in 57 patients (28.3%). A 12-month follow-up was completed by all the patients. As shown in Table 1, patients with LRAF were significantly more likely to have a history of ERAF, coronary artery disease, and higher CHA2DS2-VASC score. Also, the patients with larger LA volume, higher number of applications, and longer procedure time showed a significantly higher risk of LRAF. Additionally, the extent of CK-MB elevation was significantly decreased in patients with LRAF compared to those without LRAF.

**Table 1:** Baseline characteristics and comparison of patients with and without LRAF following catheter ablation.

Parameter	( +) LRAF (n = 57)	Lack of LRAF (n = 144)	*p* value	Total (n = 201)
Age, years, mean ± SD	64 ± 8	63 ± 11	0.43	63 ± 11
Male sex, n (%)	34 (60)	94 (65)	0.56	128 (64)
Smoking, n (%)	8 (14)	10 (7)	0.19	18 (9)
BMI, kg/${\mathrm{m}}^{2}$, mean ± SD	31 ± 7.4	30.5 ± 7.4	0.59	30.6 ± 7.3
Ablation procedure				
ERAF, n (%)	32 (56)	15 (10)	** < 0.01**	47 (23)
Procedure time, min, mean ± SD	145 ± 80	110 ± 95	**0.03**	120 ± 90
Cryoablation time, min, mean ± SD	105 ± 40	90 ± 35	**0.03**	95 ± 38.8
RF ablation time, min, mean ± SD	180 ± 80	190 ± 40	0.45	190 ± 50
Fluoroscopic time, min, mean ± SD	12.2 ± 8.6	10.7 ± 7.1	0.09	10.9 ± 7.8
Application time, min, mean ± SD	38.1 ± 24.6	32.8 ± 30.3	0.12	33.3 ± 28.4
Number of applications, mean ± SD	12 ± 9	9 ± 9	**0.05**	10 ± 9.2
Cryoablation, n (%)	29 (51)	93 (65)	0.1	122 (61)
RF ablation, n (%)	28 (49)	51 (35)	0.1	79 (39)
Cardiovascular parameters				
Baseline LA volume, ml	97 ± 43	89.4 ± 41.1	**0.02**	93 ± 40.4
CHA2DS2-VASC score, mean ± SD	3 ± 1	3 ± 2	**0.02**	3 ± 2
HAS-BLED score, mean ± SD	1 ± 1	1 ± 1	0.1	1 ± 1
SBP, mmHg, mean ± SD	130 ± 11	130 ± 17.2	0.94	130 ± 16
DBP, mmHg, mean ± SD	80 ± 10	80 ± 15	0.91	80 ± 15
Comorbities and medications				
Hypertension, n (%)	40 (70)	103 (72)	0.99	143 (71)
CAD, n (%)	17 (30)	19 (13)	**0.01**	36 (18)
Heart Failure, n (%)	6 (11)	13 (9)	0.95	19 (9)
Diabetes, n (%)	8 (14)	20 (14)	1	28 (14)
Hyperthyroidism, n (%)	7 (12)	7 (5)	0.07	14 (7)
Beta Blocker, n (%)	52 (91)	120 (83)	0.23	172 (86)
CCB, n (%)	10 (18)	32 (22)	0.59	42 (21)
NOAC, n (%)	40 (70)	103 (73)	0.74	143 (72)
VKA, n (%)	17 (30)	39 (27)	0.74	56 (28)
Statins, n (%)	47 (82)	98 (68)	0.06	145 (72)
Diuretics, n (%)	21 (37)	47 (33)	0.69	68 (34)
ACEI, n (%)	22 (39)	55 (38)	1	77 (38)
ARBs, n (%)	15 (26)	36 (25)	0.99	51 (25)
Laboratory findings				
Cholesterol, mg/dl, mean ± SD	172.5 ± 34.8	183.5 ± 48.1	0.21	180.5 ± 45.1
LDL, mg/dl, mean ± SD	106.3 ± 33.3	116.7 ± 43.2	0.19	113.8 ± 41
eGFR, ml/min, mean ± SD	73 ± 18	72 ± 20.5	0.87	72.5 ± 19.2
CRP, ug/ml, before ablation	0.2 ± 0.2	0.2 ± 0.2	0.7	0.2 ± 0.2
CRP, ug/ml, after ablation	1.0 ± 1.0	0.8 ± 0.8	0.25	0.8 ± 0.9
Δ CRP, ug/ml, mean ± SD	0.7 ± 0.9	0.5 ± 0.7	0.26	0.6 ± 0.7
PLT, 103/ml, before ablation	202 ± 54	210 ± 57.5	0.29	208 ± 59
PLT, 103/ml, after ablation	174 ± 47	179 ± 49.5	0.31	177 ± 47.2
Δ PLT, ${10}^{3}$/ml, mean ± SD	− 27 ± 25	− 31 ± 32	0.68	− 30 ± 30.2
Fibrinogen, mg/dl, before ablation	390 ± 98	379.5 ± 85.5	0.73	380.5 ± 91.5
Fibrinogen, mg/dl, after ablation	382.3 ± 72.4	368.8 ± 72.3	0.24	372.6 ± 72.6
Δ Fibrinogen, mg/dl, mean ± SD	− 8 ± 71.5	− 12 ± 55.2	0.28	− 10 ± 61.2
D-Dimer, mg/dl, before ablation	0.2 ± 0.2	0.2 ± 0.2	0.97	0.2 ± 0.2
D-Dimer, mg/dl, after ablation	0.3 ± 0.2	0.3 ± 0.2	0.29	0.3 ± 0.2
Δ D-Dimer, mg/dl, mean ± SD	0.1 ± 0.1	0 ± 0.1	0.22	0.1 ± 0.2
Hs-TnT, ng/l, before ablation	0 ± 0	0 ± 0	0.61	0 ± 0
Hs-TnT, ng/l, after ablation	0.9 ± 0.7	1.1 ± 0.6	0.13	1 ± 0.7
Δ Hs-TnT, ng/l, mean ± SD	0.9 ± 0.7	1 ± 0.6	0.13	1 ± 0.6
CPK, U/l, before ablation	113 ± 81	107.5 ± 69.5	0.36	109 ± 80
CPK, U/l, after ablation	209 ± 122	236.5 ± 151.2	0.43	222 ± 148
Δ CPK, U/l, mean ± SD	96.7 ± 132.4	115.9 ± 113.7	0.31	110.4 ± 119.6
CK-MB, U/l, before ablation	16 ± 7	15 ± 7	0.31	15 ± 6
CK-MB, U/l, after ablation	24 ± 15	28 ± 21	0.08	27 ± 20
Δ CK-MB, U/l, mean ± SD	8 ± 12	12 ± 19	**0.02**	10 ± 20

Continuous data of normal distribution are presented as mean ± standard deviation (SD).Continuous data of non-normal distribution are presented as mean IQR calculated as Q1–Q3. Categorical variables are presented as numbers and percentages. Categorical data are presented as counts with their percentage values in brackets. *p* values from the Student’s t-test are reported for normal variables, *p* values from the Mann–Whitney test are reported for non-normally distributed variables. *p* Values from the Pearson chi square test for independence are reported for categorical variables.

*BMI* body mass index, *LRAF* late recurrence atrial fibrillation, *ERAF* early recurrence atrial fibrillation, *RF* radiofrequency, *LA volume* left atrial volume, *CHA2DS2-VASc* congestive heart failure, hypertension, Age ≥ 75 (doubled), diabetes, stroke (doubled), vascular disease, age 65–74, sex (female), *HAS-BLED* hypertension, abnormal renal/liver function, stroke, bleeding history or predisposition, labile INR, elderly (> 65 years), drugs/alcohol concomitantly, *SBP* systolic blood pressure, *DBP* diastolic blood pressure, *CAD* coronary artery disease, *CCB* calcium channel blockers, *NOAC* non-vitamin K antagonist oral anticoagulant, *VKA* vitamin K antagonist, *ACE-I* angiotensin converting enzyme inhibitor, *ARB* angiotensin II receptor blocker, *GFR* glomerular filtration rate, *CRP* C-reactive protein, *PLT* platelets, *hs-TnT* high-sensitive cardiac troponin T, *CPK* creatine kinase, *CK-MB* creatine kinase-MB fraction; **Δ**—Delta denotes the response to the ablation procedure. Delta was defined as the change in the biomarker concentration between two assays performed within 24-h period (after ablation – before ablation). Significance difference LRAF(+) versus LRAF (−).

Significant values are in bold.

Table 2 shows the comparative analysis of the performance of selected classifiers. As observed, XGBoost with 12 variables achieved the highest F1 score of 0.547.

**Table 2:** Performance comparison of machine learning models: random forest (RF), XGBoost (XGB), decision trees (DT), logistic regression (LogR), and support vector machines (SVM) for a varying number of features selected with permutation importance.

Metric	# of features	DT	LogR	RF	XGB	SVM
F1	8	0.489 ± 0.138	0.56 ± 0.13	0.429 ± 0.151	0.546 ± 0.127	0.361 ± 0.114
F1	12	0.502 ± 0.134	0.529 ± 0.123	0.316 ± 0.162	**0.547 ± 0.135**	0.409 ± 0.1
F1	16	0.493 ± 0.155	0.514 ± 0.13	0.31 ± 0.168	0.54 ± 0.134	0.483 ± 0.063
F1	20	0.49 ± 0.149	0.514 ± 0.125	0.214 ± 0.167	0.514 ± 0.145	0.483 ± 0.064
F1	All	0.421 ± 0.13	0.374 ± 0.117	0.064 ± 0.107	0.433 ± 0.145	0.487 ± 0.061

Performance is measured with F1 ± standard deviation (SD) for the test set.

Parameters of machine learning methods: RF, DT, SVM: class_weight = ‘balanced’, XGB: scale_pos_weight = counts[class1]/counts[class2]; LogR: max_iter = 10,000.

Significant values are in bold.

**Figure 1:**
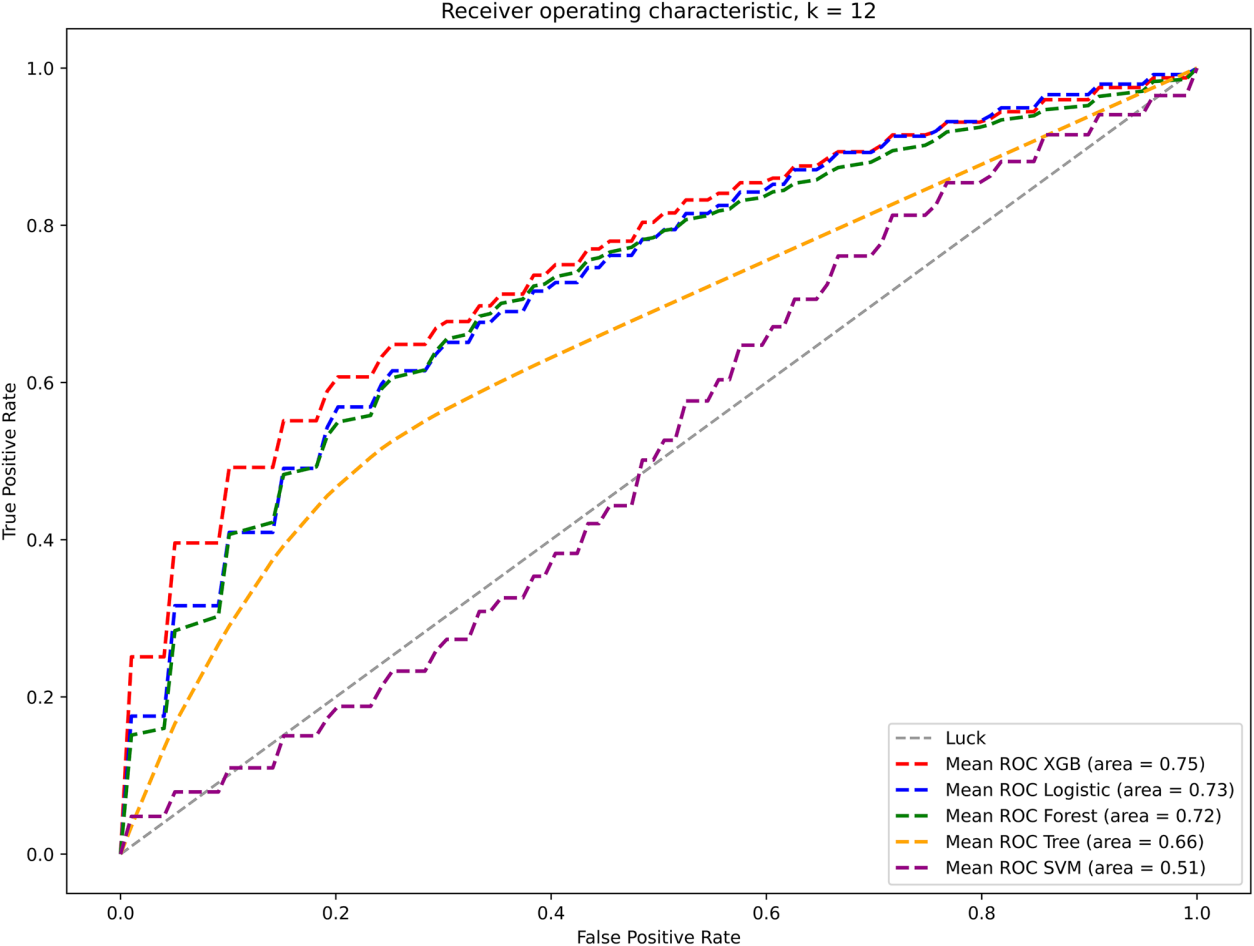
Performance of selected classifiers with 12 features validated for the validation set is further illustrated with receiver operator characteristic (ROC) curves.

The respective receiver operating characteristic (ROC) curves for this model calculated for the validation set are presented in Fig. 1. It is observed that the XGBoost model with the 12 variables achieves the highest area under the curve (AUC) of 0.75.

**Figure 2:**
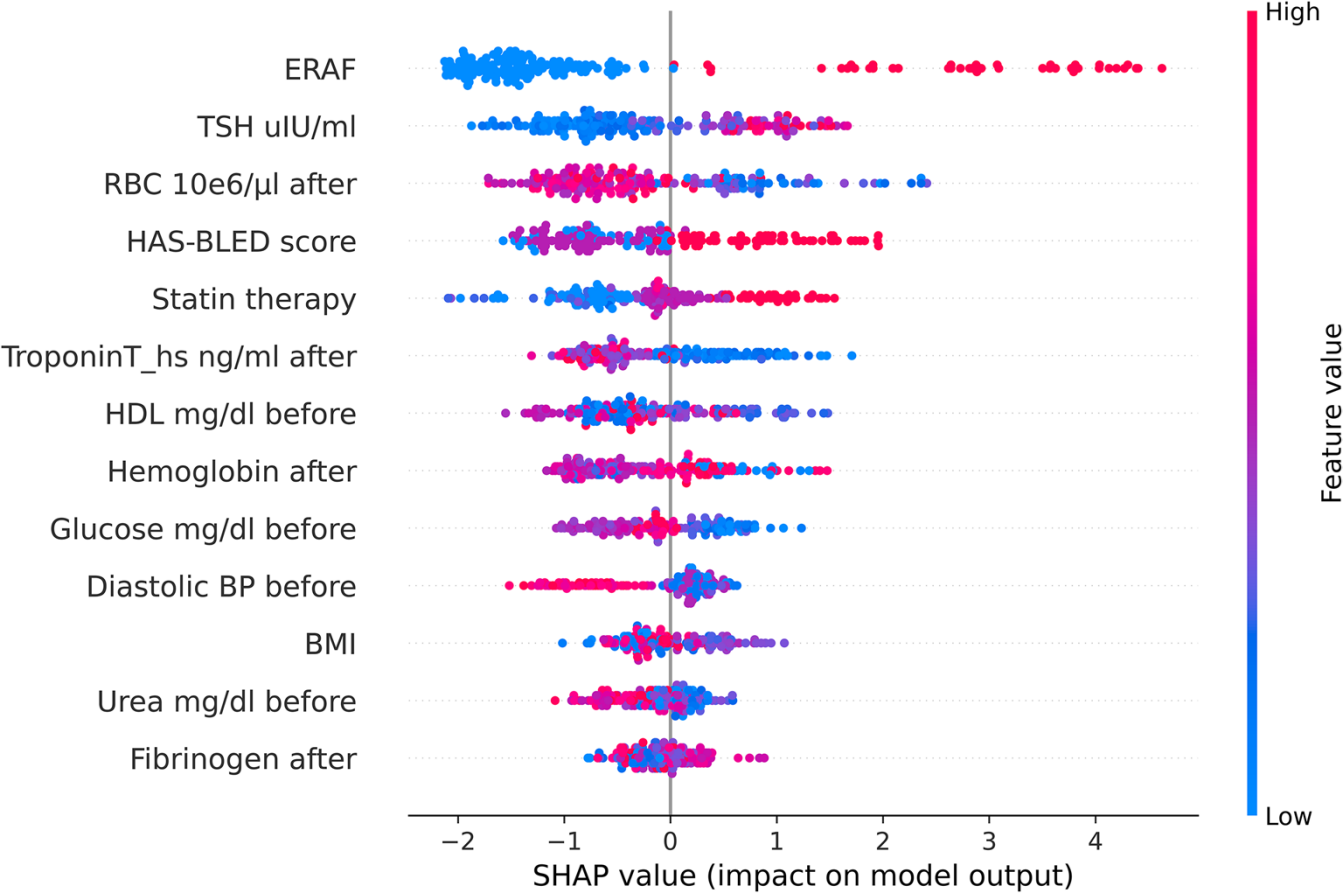
The SHAP summary plot from the XGB classifier shows the ranking of the top-most contributing features. The positive contribution towards that class is shown on the positive side of the X-axis (representing positive SHAP values), while the negative side of the axis represents a negative contribution or the contribution of those features against the prediction of that class. The XGBoost model, data samples and running examples in the Python programming language are available through the GitHub platform (https://github.com/kasiakaczmarek/predicting-late-recurrence-of-atrial-fibrillation).

The variables selected as most discriminative in this model are the following: ERAF, TSH, RBC, HAS-BLED score, BMI, statin therapy, parameters measured prior to ablation such as glucose, diastolic blood pressure, and urea, as well as parameters measured following ablation such as high-sensitive Troponin T, hemoglobin, and fibrinogen. Figure 2 shows the SHAP (SHapley Additive exPlanations) global explanation (summary) plot. Each point in the figure represents a classified data point, and the color code represents its range of feature values. SHAP presents the model output for a given class (here LRAF prediction) as an inverted pyramid of the most contributing features to that class. The high values of the top 2 features, ERAF and TSH, contribute to predicting LRAF most, while low values of the top 3 features, RBC, hsTroponin T following the procedure, and HDL cholesterol prior ablation, contribute positively to this class.

**Figure 3:**
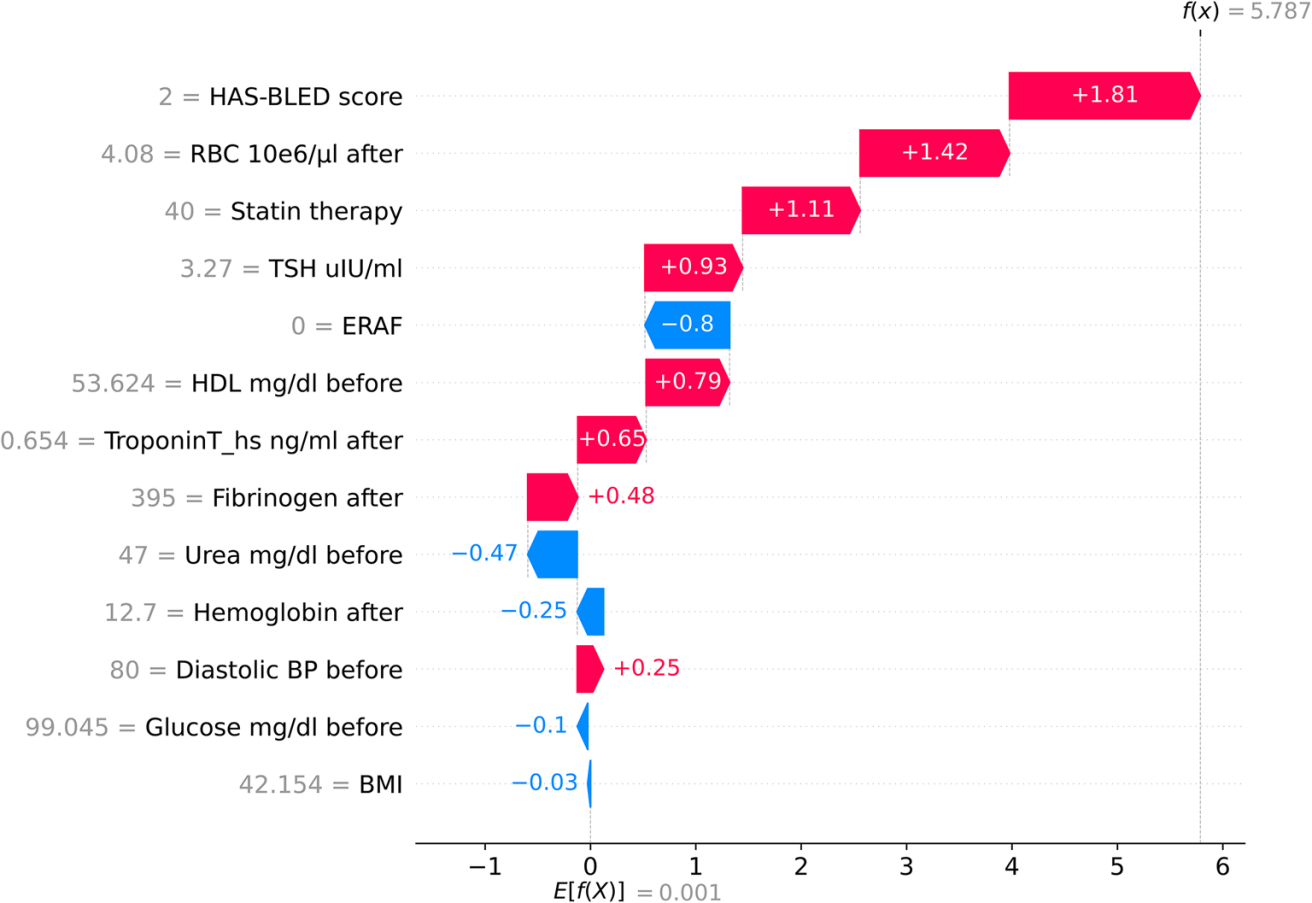
SHAP local explanations from XGB for an illustrative patient from the validation set classified as a false positive (FP) patient are shown in this figure. The red arrows represent the features that drive the prediction towards LRAF, while the blue arrows represent the features that drive the prediction against it.

Figure 3 explains in detail the prediction from the considered XGB model for an illustrative patient from the validation set classified as a false positive (FP) patient. The red arrows represent the features that drive the prediction towards LRAF, while the blue arrows represent the features that drive the prediction against it. It is observed that a higher HAS-BLED score, lower RBC levels, statin therapy, and higher TSH are the factors that increased the risk of LRAF diagnosis.

**Figure 4:**
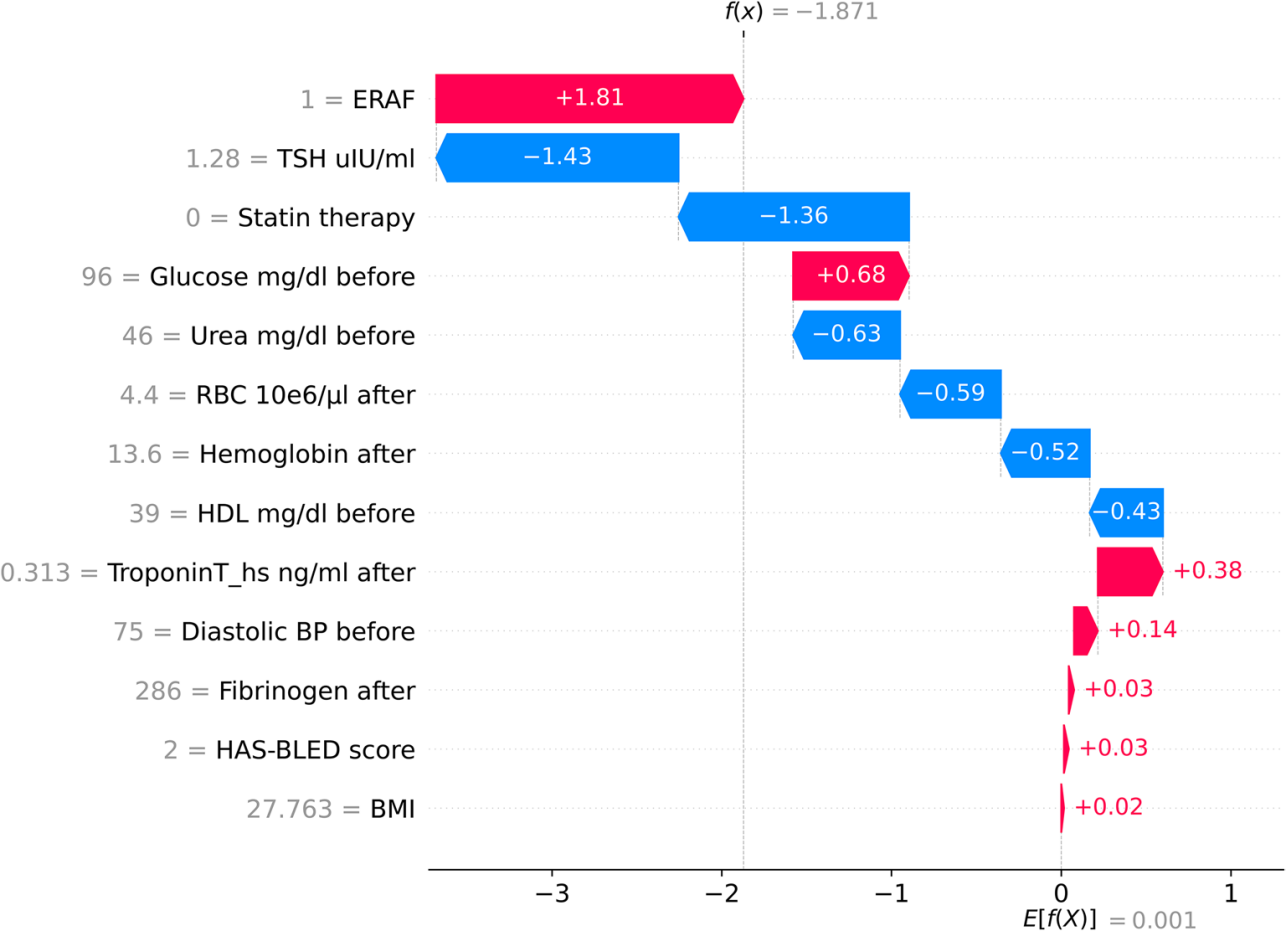
SHAP local explanations from XGB for an illustrative patient from the validation set classified as a false negative (FN) patient. The red arrows represent the features that drive the prediction towards LRAF, while the blue arrows represent the features that drive the prediction against it.

Finally, in Fig. 4, we explain in detail the prediction from the considered XGB model for an illustrative patient from the validation set classified as a false negative (FN) patient. It is observed that in this example, lower TSH values, lack of statin therapy, higher RBC and hemoglobin levels following ablation are the factors that decreased the risk of LRAF diagnosis, even despite the occurrence of ERAF.

## Discussion

Late recurrence of atrial fibrillation (LRAF) is a common phenomenon after pulmonary vein isolation, and the prognosis after the procedure is highly complex. Previous studies have demonstrated that machine learning techniques can be effectively applied for AF recurrence prediction and may have better performance than conventional statistical analysis^14^. In a recent systematic review of 33 studies developing or validating 13 models based on the c-statistic, highly variable discriminatory ability was observed, ranging from very poor to very good^13^. However, the risk of bias was high, and many studies lacked internal validation in model development.

In this study, we developed a machine learning model for predicting AF recurrence following catheter ablation in the first year after the procedure. In the examined group of 201 patients, LRAF occurred in 28% of them, with comparable frequency in both types of ablation. The proposed XGBoost model showed better performance in predicting LRAF compared to our previous model for ERAF^9^.

The XGBoost model with 12 variables commonly available in clinical practice showed the best performance on the testing cohort. As illustrated in Fig. 2, ERAF was the most important factor in the model. In addition, the SHAP results demonstrated that higher values of TSH, HAS-BLED score, statin therapy, fibrinogen, lower values of parameters measured after ablation such as RBC, troponin, hemoglobin, as well as lower values of parameters measured before ablation such as HDL, glucose, diastolic blood pressure, BMI and urea were associated with an increased risk of LRAF. Our observations show that ERAF is the factor that strongly predisposes patients to LRAF, as it comprises the most important contribution to the model.

Several other studies have reported ERAF as a very strong predictor of LRAF, both after single and multiple procedures^10,17,18^. Moreover, it has been proven that the cause of ERAF is not only the inflammatory process and tissue necrosis, but also reconnections within the pulmonary veins^18^. Therefore, Kim et al. suggest that ERAF may be a surrogate marker of the severity of AF itself and should not be considered as a transient phenomenon, but as a strong predictor of LRAF^10^. Thyroid disorders are increasingly recognized as a factor responsible for AF^19^. In the study of Morishima et al., hypothyroidism and high-normal TSH levels were independent predictors of atrial tachyarrhythmia recurrence following catheter ablation^20^. In the study of Kim et al., the hemoglobin level was also significantly lower (*p* < 0.001), and anemia was more commonly found (*p* < 0.001) in patients with clinical recurrence following ablation than in those who remained in sinus rhythm^21^. On the other hand, the HAS-BLED score, as a predictor of bleeding adverse events, also includes important risk factors for AF recurrence, such as hypertension and advanced age.

### Study limitations

The study was single-centered with a relatively small number of patients. The study group was heterogeneous in terms of the number of ablation procedures and RF ablation technique. LRAF was detected based on clinical symptoms, 12-lead ECG, and 24-h Holter monitoring. Therefore, asymptomatic ERAF might have been missed in outcome adjudication. Also, a specific limitation in the interpretation of myocardial injury biomarkers, such as CPK and CK-MB, occurred due to their thermal instability during RF ablation^22^. Finally, the main limitation of machine learning analysis was the small data set.

## Conclusions

Our machine learning model of LRAF following catheter ablation achieved good performance and works as a valuable tool for better patient qualification.

### Supplementary Information

Supplementary Information 1.

Supplementary Information 2.

## Data Availability

The datasets used and/or analyzed in this study are available from the corresponding authors upon reasonable request. The XGBoost model, data samples and running examples are available through the GitHub platform (https://github.com/kasiakaczmarek/predicting-late-recurrence-of-atrial-fibrillation). Full dataset used to generate these results is subject to data sharing agreements and cannot be made publicly available but can be shared following the agreement of respective institutional review boards on request to the corresponding author.
