# Supplementary material for: Machine learning model for predicting late recurrence of atrial fibrillation after catheter ablation
Source: Sci Rep. 2023 Sep 14;13:15213. doi: 10.1038/s41598-023-42542-y (PMC10502018; doi:10.1038/s41598-023-42542-y)
Supplement: Supplementary file 1 — Supplementary Information 1. [file 41598_2023_42542_MOESM1_ESM.docx]

**Table S1.** Description of all clinical and laboratory variables included in this study. Variables marked in bold were selected by the XGBoost model presented in this paper.

| **Variable** | **Type** | **Definition and methodological comments** |
| --- | --- | --- |
| Age (years) | Clinical | Age at the time of inclusion in the study is considered as a demographic patient characteristic |
| Smoking, n (%) | Clinical | Smoking of loose tobacco or cigarettes |
| **BMI, kg/m2** | **Clinical** | **Body mass index (BMI) defined as a person’s weight divided by their height in meters squared (expressed in kg/m^2^)** |
| LRAF | Target variable | Late recurrence of atrial fibrillation (LRAF) defined as any symptomatic or asymptomatic atrial tachyarrhythmias (AF, atrial tachycardia [AT], or atrial flutter [AFL]) lasting > 30 s) that occurred 3 months (up to 1 year) following the procedure |
| **ERAF, n (%)** | **Clinical** | **Early recurrence of atrial fibrillation (ERAF) defined as at least a 30-second episode of arrhythmia (AF, atrial tachycardia [AT], or atrial flutter [AFL]) registered by a 24h-Holter monitor within the first 3 months following the procedure** |
| Procedure time, min | Procedural | Procedure time defined as the duration of cryoballoon and radiofrequency (RF) ablation |
| Cryoablation time, min | Procedural | Defined as the duration of cryoballoon ablation |
| RF ablation time, min | Procedural | Defined as the duration of RF ablation |
| Fluoroscopic time, min | Procedural | Defined as the time of fluoroscopy during cryoballoon and RF ablation |
| Application time, min | Procedural | Defined as the time of application during cryoballoon and RF ablation |
| Number of applications | Procedural | Defined as the number of cryoballoon and RF applications |
| Cryoablation, n (%) | Procedural | The number of cryoballoon ablation procedures (expressed in percentage) |
| RF ablation, n (%) | Procedural | The number of RF ablation procedures (expressed in percentage) |
| LA volume, ml | +Echocardiography | Left atrial volume calculated from biplane recordings during transthoracic echocardiography |
| CHA2DS2-VASC score | Clinical | The most popular method to predict thromboembolic risk in AF. Congestive heart failure, Hypertension, Age ≥ 75 (doubled), Diabetes, Stroke (doubled), Vascular disease, Age 65–74, Sex (female). Minimum score is 0 (male) or 1 (female). Maximum score is 9 |
| **HAS-BLED score** | **Clinical** | **A scoring system to estimate the bleeding risk in patients with AF. Hypertension, Abnormal renal/liver function, Stroke, Bleeding history or predisposition, Labile INR, Elderly (>65 years), Drugs/alcohol concomitantly. A calculated HAS-BLED score is between 0 and 9 and based on seven parameters with a measured value of 0-2** |
| SBP, mmHg | Clinical | Systolic blood pressure (SBP) defined as the maximum blood pressure during contraction of the ventricles measured prior to and following catheter ablation |
| **DBP, mmHg** | **Clinical** | **Diastolic blood pressure (DBP) is the minimum pressure recorded prior to the next contraction. DBP was measured before and after catheter ablation** |
| Hypertension, n (%) | Comorobities | Hypertension defined as 1 when blood pressure values surpassed 140/90 mmHg; otherwise considered as 0 |
| CAD, n (%) | Comorobities | Coronary artery disease (CAD) comprises a spectrum of clinical syndromes caused by insufficient coronary blood flow to myocardium |
| Heart Failure, n (%) | Comorobities | Heart failure (HF) is a complex clinical syndrome that can result from any structural or functional cardiac disorder impairing the ability of the ventricle to absorb or eject blood. |
| Diabetes, n (%) | Comorobities | Diabetes is a chronic metabolic disease characterized by elevated levels of glucose in the blood. If the patients met the criteria for diabetes, they were marked as 1; otherwise considered as 0  Criteria for the diagnosis of diabetes: 1) HbA1c ≥6.5% or 2) fasting plasma glucose (FPG) ≥126 mg/dL (7 mmol/L), or 3) 2-hour plasma glucose ≥200 mg/dL (11.1 mmol/L) during an OGTT.or 4) In a patient with classic symptoms of hyperglycemia or hyperglycemic crisis, a random plasma glucose ≥200 mg/dL (11.1 mmol/L). |
| Hyperthyroidism, n (%) | Comorobities | Hyperthyroidism is the condition that occurs due to the excessive production of thyroid hormones by the thyroid gland |
| Beta Blocker, n (%) | Medications | Beta blockers are medications that have beta-adrenergic blocking properties, meaning that they block the sympathetic nervous system activity |
| CCB, n (%) | Medications | Calcium channel blockers (CCB) are the class of drugs that act by selective inhibition of calcium influx through cell membranes or on the release and binding of calcium in intracellular pools. Since they are inducers of vascular and other smooth muscle relaxation, they are used in the drug therapy of hypertension |
| NOAC, n (%) | Medications | Non–vitamin K antagonist oral anticoagulants (NOAC) termed as direct oral anticoagulants or target anticoagulants due to their direct inactivation of thrombin (FIIa) and factor X (FXa) |
| VKA, n (%) | Medications | Vitamin K antagonists (VKAs) are coumarin drugs that interfere in the carboxylation of vitamin K-dependent factors (factor VII, IX, X and prothrombin) and anticoagulation proteins C and S |
| **Statin therapy, n (%)** | **Medications** | **[Statins](https://www.sciencedirect.com/topics/pharmacology-toxicology-and-pharmaceutical-science/hydroxymethylglutaryl-coenzyme-a-reductase-inhibitor), inhibitors of the hydroxymethylglutaryl-CoA (HMG-CoA) [reductase](https://www.sciencedirect.com/topics/pharmacology-toxicology-and-pharmaceutical-science/oxidoreductase) enzyme act as cholesterol lowering medications. Statin therapy was marked as 1 when the patients had taken statins before admission to hospital;** otherwise considered as 0 |
| Diuretics, n (%) | Medications | Diuretics are medications that induce kidneys to increase urine production |
| ACEI, n (%) | Medications | Angiotensin-converting-enzyme inhibitors (ACE inhibitors) are a class of medication used primarily to treat high blood pressure and heart failure |
| ARBs, n (%) | Medications | Angiotensin receptor blockers (ARBs) are medication used to treat high blood pressure and heart failure |
| TSH, uIU/ml | Laboratory | Thyroid-stimulating hormone, also known as TSH, is a glycoprotein hormone produced by the anterior pituitary. It stimulates the thyroid gland to produce hormones. |
| Cholesterol before, mg/dl | Laboratory | The complete amount of low-density lipoprotein cholesterol (), high-density lipoprotein cholesterol (HDL), and very low-density lipoprotein cholesterol (VLDL) |
| LDL before, mg/dl | Laboratory | Low-density lipoprotein cholesterol. is the major cholesterol-carrying particle in plasma. It is an independent risk factor for artherosclerosis. It is measured before catheter ablation. |
| **HDL before, mg/l** | **Laboratory** | **High-density lipoprotein (HDL) is known for its anti-atherogenic and anti-inflammatory properties. It is measured before catheter ablation** |
| TG mg/dl before | Laboratory | A triglyceride (TG) molecule consists of a glycerol backbone esterified with three fatty acids. |
| eGFR, ml/min | Laboratory | Estimated Glomerular filtration rate (GFR) is defined as the volume of plasma that is filtered by the glomeruli per unit of time. GFR was measured by MDRD formula |
| Creatinine | Laboratory | Creatinine is a metabolic product of creatine and [phosphocreatine](https://www.sciencedirect.com/topics/medicine-and-dentistry/creatine-phosphate), both of which are found almost exclusively in muscles. It is measured before catheter ablation |
| **Urea before, mg/dl** | **Laboratory** | **Commonly referred to as blood urea nitrogen (BUN) when measured in the blood. It is a product of protein metabolism, which is measured before catheter ablation** |
| Δ CRP, ug/ml | Laboratory | A C-reactive protein (CRP) is one of t plasma proteins known as acute-phase protein of hepatic origin that increases in response to inflammation. Delta was defined as the change in the biomarker concentration between two assays performed within a 24-hour period (after ablation – before ablation) |
| CRP before, ug/ml, | Laboratory | C-reactive protein measured before catheter ablation |
| CRP after, ug/ml | Laboratory | C-reactive protein measured 24 hours after catheter ablation |
| Δ PLT, 103/ml | Laboratory | Platelets (PLT) are essential for primary haemostasis and form a temporary haemostatic plug prior to the activation of the clotting cascade. The PLT is the number of platelets in a sample of blood. Delta was defined as the change in the biomarker concentration between two assays performed within a 24-hour period (after ablation – before ablation). |
| PLT, 10e3/ml, before | Laboratory | Platelet count measured before catheter ablation |
| PLT, 10e3/ml, after | Laboratory | Platelet count measured 24 hours after catheter ablation |
| WBC 10e3/ml | Laboratory | Leukocytes originate from bone marrow and consist of granulocytic and mononuclear cells. These cells are important in both the innate and adaptive immune response. They are measured before and after catheter ablation |
| RDW, % | Laboratory | Red cell distribution width (RDW) refers to the variation degree of [erythrocyte size](https://www.sciencedirect.com/topics/medicine-and-dentistry/erythrocyte-volume) and it is a reflection of [anisocytosis](https://www.sciencedirect.com/topics/medicine-and-dentistry/anisocytosis). Measured before and after catheter ablation |
| MCHC, g/dl | Laboratory | [Mean corpuscular hemoglobin](https://www.sciencedirect.com/topics/biochemistry-genetics-and-molecular-biology/mean-corpuscular-hemoglobin) concentration (MCHC) is the average hemoglobin concentration per RBC. Measured before and after catheter ablation. |
| MCH, pg | Laboratory | Mean corpuscular hemoglobin (MCH) levels refer to the average amount of hemoglobin found in red blood cells |
| Δ Fibrinogen, mg/dl | Laboratory | Fibrinogen hexameric plasma glycoprotein synthesized by liver forms the major structural component of a clot. Delta was defined as the change in the biomarker concentration between two assays performed within a 24-hour period (after ablation – before ablation). |
| Fibrinogen, mg/dl, before | Laboratory | Fibrinogen measured before catheter ablation |
| **Fibrinogen, mg/dl, after** | **Laboratory** | **Fibrinogen measured 24 hours after catheter ablation** |
| Δ D-Dimer, mg/dl | Laboratory | D-dimer is an indirect marker of fibrinolysis and fibrin turnover. Delta was defined as the change in the biomarker concentration between two assays performed within a 24-hour period (after ablation – before ablation). |
| D-Dimer, mg/dl, before | Laboratory | D-dimer measured before catheter ablation |
| D-Dimer, mg/dl, after | Laboratory | D-dimer measured 24 hours after catheter ablation |
| Δ TroponinT_hs, ng/l | Laboratory | High-sensitivity troponins (TroponinT_hs) are essential for the interpretation and diagnosis of either myocardial infarction or myocardial injury. Delta was defined as the change in the biomarker concentration between two assays performed within a 24-hour period (after ablation – before ablation). |
| TroponinT_hs before ng/l, | Laboratory | Hs-Troponin T measured before catheter ablation |
| **TroponinT_hs after ng/l,** | **Laboratory** | **Hs-Troponin T measured 24 hours after catheter ablation** |
| Δ CPK, U/l | Laboratory | Creatine phosphokinase (CPK) also known as Creatine kinase (CK) is an enzyme that catalyzes the phosphorylation of creatine. CPK is an [intracellular enzyme](https://www.sciencedirect.com/topics/pharmacology-toxicology-and-pharmaceutical-science/cell-enzyme) mostly present in skeletal muscles, myocardium, and brain; smaller amounts occur in other visceral tissues. Delta was defined as the change in the biomarker concentration between two assays performed within a 24-hour period (after ablation – before ablation). |
| CPK before, U/l | Laboratory | Creatine phosphokinase measured before catheter ablation |
| CPK after, U/l | Laboratory | Creatine phosphokinase measured 24 hours after catheter ablation |
| Δ CK-MB, U/l | Laboratory | Creatine Kinase isoenzyme MB is one of the three forms (or isoenzymes) of the creatine kinase enzyme (CK). CK–MB is found mostly in heart muscles. Delta was defined as the change in the biomarker concentration between two assays performed within a 24-hour period (after ablation – before ablation). |
| CK-MB before, U/l | Laboratory | CK-MB measured before catheter ablation |
| CK-MB after, U/l | Laboratory | CK-MB measured 24 hours after catheter ablation |
| Δ Hemoglobin , mg/dl | Laboratory | Hemoglobin is a protein contained in red blood cells that is responsible for delivery of oxygen to the tissues. Normal range: 11.2 – 15.7 g/dl. Delta was defined as the change in the biomarker concentration between two assays performed within a 24-hour period (after ablation – before ablation). |
| Hemoglobin before , mg/d | Laboratory | Hemoglobin measured before catheter ablation |
| **Hemoglobin after , mg/d** | **Laboratory** | **Hemoglobin measured 24 hours after catheter ablation** |
| Δ RBC 10e3/ul | Laboratory | Red blood cells (RBCs, erythrocytes) are highly specialized cells responsible for the transport of respiratory gases. Delta was defined as the change in the biomarker concentration between two assays performed within a 24-hour period (after ablation – before ablation). |
| RBC 10e3/ul before | Laboratory | RBC measured before catheter ablation |
| **RBC 10e3/ul after** | **Laboratory** | **RBC measured 24 hours after catheter ablation** |
| **Glucose before, mg/dl** | **Laboratory** | **A fasting plasma glucose concentration measured before catheter ablation. Normal range: 65-99 mg/dl.** |
| INR before | Laboratory | International normalized ratio (INR) is typically used to monitor patients in an oral anticoagulant therapy (warfin , acenokumarol). Measured before catheter ablation |
| APTT before, sec | Laboratory | Activated partial thromboplastin time (aPTT) is a coagulation screening assay that estimates the activity of the intrinsic and common coagulation pathway. Measured before catheter ablation |
| ALT before, U/l | Laboratory | Alanine aminotransferase (ALT) catalyzes the transfer of an amino group from [alanine](https://www.sciencedirect.com/topics/biochemistry-genetics-and-molecular-biology/alanine) to alpha-ketoglutarate in the alanine cycle to form pyruvate and glutamate. Measured before catheter ablation |
| AST before, U/l | Laboratory | Aspartate aminotransferase (AST) is a transaminase [enzyme](https://www.sciencedirect.com/topics/biochemistry-genetics-and-molecular-biology/lysozyme) that catalyzes the conversion of aspartate and alpha-ketoglutarate to oxaloacetate and glutamate. Measured before catheter ablation |
| Sodium before, mmol/l | Laboratory | Sodium is the major cation of extracellular fluid. Sodium is essential for cellular homeostasis and physiological function. Measured before catheter ablation. |
| Potassium before,mmol/l | Laboratory | Potassium is the main intracellular cation in the body and is principally involved in forming the membrane potential. Measured before catheter ablation |
