# Supplementary material for: Machine learning model for predicting late recurrence of atrial fibrillation after catheter ablation
Source: Sci Rep. 2023 Sep 14;13:15213. doi: 10.1038/s41598-023-42542-y (PMC10502018; doi:10.1038/s41598-023-42542-y)
Supplement: Supplementary file 2 — Supplementary Information 2. [file 41598_2023_42542_MOESM2_ESM.docx]

Table S2. Additional laboratory data of patients with and without LRAF following catheter ablation.

| **LABORATORY FINDINGS** | | | |
| --- | --- | --- | --- |
| **Parameter** | **(+) LRAF (n=57 )** | **Lack of LRAF (n=144)** | **Total (n=201)** |
| WBC before,${10}^{3}$/ml , mean ± SD | 7.25 ± 1.68 | 6.78 ± 1.69 | 7.03 ± 1.71 |
| WBC after, ${10}^{3}$/ml , mean ± SD | 9.14 ± 2.51 | 8.50 ± 2.01 | 9.08 ± 2.56 |
| RBC before,${10}^{6}$/ml , mean ± SD | 4.79 ± 0.62 | 4.84 ± 0.45 | 4.82 ± 0.58 |
| RBC after, ${10}^{6}$/ml , mean ± SD | 4.30 ± 0.61 | 4.47 ± 0.51 | 4.35 ± 0.52 |
| Hemoglobin before g/dl, mean ± SD | 14,85 ± 1,59 | 15.18 ± 1.23 | 15.01 ± 1.36 |
| Hemoglobin after g/dl, mean ± SD | 13.38 ± 1.85 | 14.02 ± 1.29 | 13.66 ± 1.53 |
| HDL mg/dl, mean ± SD | 57.86 ± 15.79 | 59.75 ± 15.90 | 58.18 ± 14.80 |
| TG mg/dl, mean ± SD | 118.32 ± 45.38 | 136.26 ± 82.08 | 123.40 ± 58.16 |
| Glucose mg/dl mean ± SD | 101.0 ± 15.33 | 103.86 ± 19.65 | 101.52 ± 16.33 |
| INR , mean ± SD | 1.60 ± 0.68 | 1.70 ± 0.70 | 1.60 ± 0.66 |
| APTT, sec, mean ± SD | 41.35 ± 9.62 | 39.98 ± 8.24 | 41.03 ± 9.16 |
| Na, mmol/l, mean ± SD | 142.00 ± 2.58 | 141.96 ± 2.13 | 142.05 ± 2.24 |
| K, mmol/l, mean ± SD | 4.55 ± 0.41 | 4.50 ± 0.36 | 4.53 ± 0.37 |
| Urea, mg/dl, mean ± SD | 36.86 ± 9.40 | 39.76 ± 9.20 | 38.23 ± 9.41 |
| Creatinine, mg/dl, mean ± SD | - 1. ± 0.22 | 1.06 ± 0.21 | 1.02 ± 0.21 |
| TSH, mg/dl, mean ± SD | 2.21 ± 2.08 | 1.92 ± 1.58 | 2.15 ± 2.06 |
| ALAT, U/l, mean ± SD | 26.46 ± 10.76 | 27.93 ± 13.63 | 26.95 ± 12.34 |
| AST, U/l mean ± SD | 23.42 ± 5.95 | 24.96 ± 7.06 | 24.17 ± 6.71 |

Continuous data of normal distribution are presented as mean ± standard deviation (SD).

Abbreviations: WBC – white blood count; RBC – red blood cells; HDL – high-density lipoprotein cholesterol; TG – triglycerides; INR – International Normalized Ratio; APTT - Active partial thromboplastin time; TSH – thyroid stimulating hormone; ALAT – Alanine aminotransferase; AST-  aspartate aminotransferase
